# Diagnostic FDG and FDOPA positron emission tomography scans distinguish the genomic type and treatment outcome of neuroblastoma

**DOI:** 10.18632/oncotarget.7933

**Published:** 2016-03-05

**Authors:** Yen-Lin Liu, Meng-Yao Lu, Hsiu-Hao Chang, Ching-Chu Lu, Dong-Tsamn Lin, Shiann-Tarng Jou, Yung-Li Yang, Ya-Ling Lee, Shiu-Feng Huang, Yung-Ming Jeng, Hsinyu Lee, James S. Miser, Kai-Hsin Lin, Yung-Feng Liao, Wen-Ming Hsu, Kai-Yuan Tzen

**Affiliations:** ^1^ Department of Pediatrics, Taipei Medical University Hospital, Taipei, Taiwan; ^2^ PhD Program in Translational Medicine, National Taiwan University and Academia Sinica, Taipei, Taiwan; ^3^ Institute of Cellular and Organismic Biology, Academia Sinica, Taipei, Taiwan; ^4^ Department of Pediatrics, National Taiwan University Hospital and National Taiwan University College of Medicine, Taipei, Taiwan; ^5^ Taipei Cancer Center, Taipei Medical University, Taipei, Taiwan; ^6^ Department of Nuclear Medicine, National Taiwan University Hospital and National Taiwan University College of Medicine, Taipei, Taiwan; ^7^ Department of Laboratory Medicine, National Taiwan University Hospital and National Taiwan University College of Medicine, Taipei, Taiwan; ^8^ Department of Nursing, National Taiwan University Hospital and National Taiwan University College of Medicine, Taipei, Taiwan; ^9^ Institute of Molecular and Genomic Medicine, National Health Research Institutes, Miaoli, Taiwan; ^10^ Department of Pathology, National Taiwan University Hospital and National Taiwan University College of Medicine, Taipei, Taiwan; ^11^ Department of Life Science, College of Life Science, National Taiwan University, Taipei, Taiwan; ^12^ College of Medical Science and Technology, Taipei Medical University, Taipei, Taiwan; ^13^ Department of Surgery, National Taiwan University Hospital and National Taiwan University College of Medicine, Taipei, Taiwan; ^14^ Research Center for Developmental Biology and Regenerative Medicine, National Taiwan University, Taipei, Taiwan; ^15^ Molecular Imaging Center, National Taiwan University, Taipei, Taiwan

**Keywords:** neuroblastoma, positron emission tomography, FDG, FDOPA, copy number alterations

## Abstract

Neuroblastoma (NB) is a heterogeneous childhood cancer that requires multiple imaging modalities for accurate staging and surveillances. This study aims to investigate the utility of positron emission tomography (PET) with ^18^F-fluorodeoxyglucose (FDG) and ^18^F-fluoro-dihydroxyphenylalanine (FDOPA) in determining the prognosis of NB. During 2007–2014, forty-two NB patients (male:female, 28:14; median age, 2.0 years) undergoing paired FDG and FDOPA PET scans at diagnosis were evaluated for the maximum standardized uptake value (SUV_max_) of FDG or FDOPA by the primary tumor. Patients with older age, advanced stages, or *MYCN* amplification showed higher FDG and lower FDOPA SUV_max_ (all *P* < 0.02). Receiver operating characteristics analysis identified FDG SUV_max_≥ 3.31 and FDOPA SUV_max_ < 4.12 as an ultra-high-risk feature (PET-UHR) that distinguished the most unfavorable genomic types, *i.e.* segmental chromosomal alterations and/or *MYCN* amplification, at a sensitivity of 81.3% (54.4%–96.0%) and a specificity of 93.3% (68.1%–99.8%). Considering with age, stage, *MYCN* status, and anatomical image-defined risk factor, PET-UHR was an independent predictor of inferior event-free survival (multivariate hazard ratio, 4.9 [1.9–30.1]; *P* = 0.012). Meanwhile, the ratio between FDG and FDOPA SUV_max_ (G:D) correlated positively with *HK2* (Spearman's *ρ* = 0.86, *P* < 0.0001) and negatively with *DDC* (*ρ* = −0.58, *P* = 0.02) gene expression levels, which might suggest higher glycolytic activity and less catecholaminergic differentiation in NB tumors taking up higher FDG and lower FDOPA. In conclusion, the intensity of FDG and FDOPA uptake on diagnostic PET scans may predict the tumor behavior and complement the current risk stratification systems of NB.

## INTRODUCTION

Neuroblastoma (NB) is an embryonal cancer originating from the sympathetic nervous system [1]. The clinical course of NB is highly variable [1]. For the purposes of staging and surveillance, a combination of bone marrow studies, urinary catecholamine levels, and multiple imaging modalities have been used to accurately define the extensiveness of disease [2].

Traditionally, the tumor biology of NB has been defined by age, stage, histopathology, *MYCN* amplification, and ploidy [1]. More recently, overall genomic patterns of copy number alterations were proven to have independent prognostic value [3, 4]. In addition, CT or MR image-defined risk factors (IDRFs) such as tumor encasement of major structures predict worse outcome in localized tumors [5]. Whether modern molecular imaging tools performed at diagnosis can help to predict tumor biology or treatment outcome has been less studied.

^123^I-Metaiodobenzylguanidine (^123^I-MIBG) scintigraphy is the standard molecular imaging of NB [6, 7]. By targeting the norepinephrine transporter (encoded by *SLC6A2*) [8], MIBG is specifically accumulated in NB cells, not only providing diagnostic and prognostic values [9, 10] but also serving as a prelude to targeted radiotherapy with ^131^I-MIBG in ultra-high-risk patients [11].

Due to its increasing availability, positron emission tomography (PET) with ^18^F-fluorodeoxyglucose (FDG) has been explored in NB [12-15]. By targeting aerobic glycolysis of cancer cells, known as the Warburg effect [16], FDG PET provides additional diagnostic information for NB that fails to accumulate or weakly condenses MIBG [12], and complements ^123^I-MIBG scan in demonstrating localized NBs and soft-tissue lesions [13]. The international guidelines for NB recommend FDG PET as an option to imaging with ^123^I-MIBG [6].

More recently, ^18^F-fluoro-dihydroxyphenylalanine (FDOPA) PET has shown high accuracy compared with CT/MR imaging [17] and good sensitivity compared with ^123^I-MIBG scintigraphy [18, 19]. By targeting the ubiquitous expression of aromatic l-amino acid decarboxylase (encoded by the *DDC* gene) in NB [20], which catalyzes l-DOPA to l-dopamine in the catecholamine biosynthesis pathway, the whole-body metabolic burden of FDOPA also confer a prognostic role in relapsed/refractory NBs [21]. However, the prognostic value of FDOPA PET scan at initial diagnosis of NB remains to be elucidated.

As the commercial supply of ^123^I-MIBG has been limited in Taiwan, we have utilized FDG and FDOPA PET in diagnosing and following NB patients. This study aims to investigate the association between the tumor uptake pattern of FDG and FDOPA on diagnostic PET scans and the clinical features, genomic types, as well as treatment outcome in NB.

## RESULTS

### Clinical and imaging features

During the study period from June 2007 to July 2014, 88 patients with clinical diagnosis of NB were enrolled for PET scans. Forty-six patients were excluded from analysis: Forty-two patients had PET scans performed after receiving the second chemotherapy cycle or during post-treatment follow-up only; two patients only had FDG PET at diagnosis; one patient only had FDOPA PET at diagnosis; one patient had gross total resection (GTR) of primary NB tumor prior to PET imaging. The rest 42 patients who had paired FDG and FDOPA imaging performed on different days at initial diagnosis were eligible for analysis (Supplementary Figure S1). There were 28 boys and 14 girls. The median age at diagnosis was 2.0 years (interquartile range [IQR], 0.5–4.9 years). Most patients were older than 18 months (*n* = 24; 57%), had stage 4 disease (*n* = 25; 60%), and belonged to the high-risk group (*n* = 30; 71%) (Table 1).

**Table 1:** Patient characteristics and tumor uptake of FDG and FDOPA

Characteristics	No.	FDG Uptake*	*P*^†^	FDOPA Uptake*	*P*^†^	G:D Ratio*	*P*^†^
**All patients**	42	4.07 (2.76–6.36)	**–**	3.54 (2.79–5.03)	–	1.40 (0.75–1.89)	–
**Gender**			0.35		0.46		0.73
Female	14	3.25 (2.49–5.64)		3.53 (2.32–4.72)		1.29 (0.75–1.75)	
Male	28	4.53 (2.84–6.99)		3.54 (2.94–6.09)		1.40 (0.66–2.25)	
**Age group**			**0.0095**		**0.01**		**0.0018**
< 18 months	18	2.83 (2.24–5.09)		5.06 (2.96–6.65)		0.79 (0.35–1.59)	
≥ 18 months	24	5.09 (3.56–7.60)		3.28 (2.59–3.70)		1.64 (1.09–2.61)	
**Stage**			**0.0069**		**0.0013**		**0.0005**
1/2/3/4S	6/1/9/1	2.77 (1.94–4.29)		5.00 (3.46–6.67)		0.61 (0.32–1.26)	
4	25	5.16 (3.40–7.31)		3.09 (2.43–3.61)		1.67 (1.04–2.56)	
***MYCN***			**0.0067**		0.11		**0.0023**
Non-amplified	33	3.79 (2.64–5.11)		3.59 (2.95–5.30)		0.99 (0.57–1.63)	
Amplified	9	6.85 (5.64–8.17)		3.24 (2.04–3.66)		1.86 (1.69–2.90)	
**Genomic type**			**0.0003**		**0.0074**		**0.0001**
Numerical	11	2.68 (2.1–3.0)		5.49 (4.12–7.34)		0.43 (0.27–0.78)	
Segmental	7	5.01 (3.48–5.76)		3.58 (3.33–3.70)		1.35 (0.99–1.61)	
*MYCN*-amplified	9	6.85 (5.64–8.17)		3.24 (2.04–3.66)		1.86 (1.69–2.90)	
Flat/IGF2^‡^	3/1	6.38 (1.73–12.45)		3.95 (2.14–6.19)		1.29 (0.51–3.63)	
**Histology**			0.70		0.95		0.67
UNB/PDNB	9/26	4.10 (3.03–6.19)		3.58 (2.85–5.00)		1.44 (0.78–1.86)	
DNB/GNBi	3/2	2.72 (1.75–11.38)		3.48 (2.39–5.76)		0.92 (0.58–2.84)	
NB, unspecified^‡^	2	–		–		–	
**Risk group**			**0.0037**		**0.0057**		**0.0008**
Low	7	2.30 (1.62–2.77)		5.49 (4.12–6.72)		0.43 (0.27–0.98)	
Intermediate	5	3.03 (2.04–7.72)		6.62 (4.06–9.10)		0.61 (0.26–1.33)	
High	30	5.11 (3.44–7.17)		3.28 (2.55–3.69)		1.64 (1.06–2.43)	
**Site**			0.97		0.69		0.78
Adrenal	29	4.05 (2.70–6.94)		3.48 (2.43–5.24)		1.49 (0.64–2.34)	
RP/Med	11/2	4.10 (3.11–5.47)		3.59 (2.91–4.61)		1.35 (0.89–1.74)	
**IDRF**			**0.03**		0.24		**0.03**
0	15	2.77 (1.80–5.60)		4.12 (2.45–6.47)		0.98 (0.22–0.97)	
≥ 1	27	4.59 (3.32–7.03)		3.41 (2.85–4.09)		1.61 (0.80–2.33)	

* Presented as median (interquartile range).

† By two-tailed Kruskal-Wallis test; *P* values in bold font indicate significance.

‡ These categories were not included in statistical analyses.

Abbreviations: CR = complete response after induction chemotherapy; DNB = differentiating neuroblastoma; GNBi, ganglioneuroblastoma, intermixed; G:D = the ratio between the SUV_max_ of FDG and FDOPA; GTR = gross total resection; IDRF, image-defined risk factor; IGF2 = Beckwith-Wiedemann syndrome with *IGF2* microdeletion; IQR = interquartile range; Med = mediastinal; NA = second operation was not attempted due to ongoing chemotherapy or disease progression; NB = neuroblastoma; PDNB = poorly-differentiated neuroblastoma; RP = retroperitoneal; UNB = undifferentiated neuroblastoma; GNBi = ganglioneuroblastoma, intermixed.

**Figure 1:**
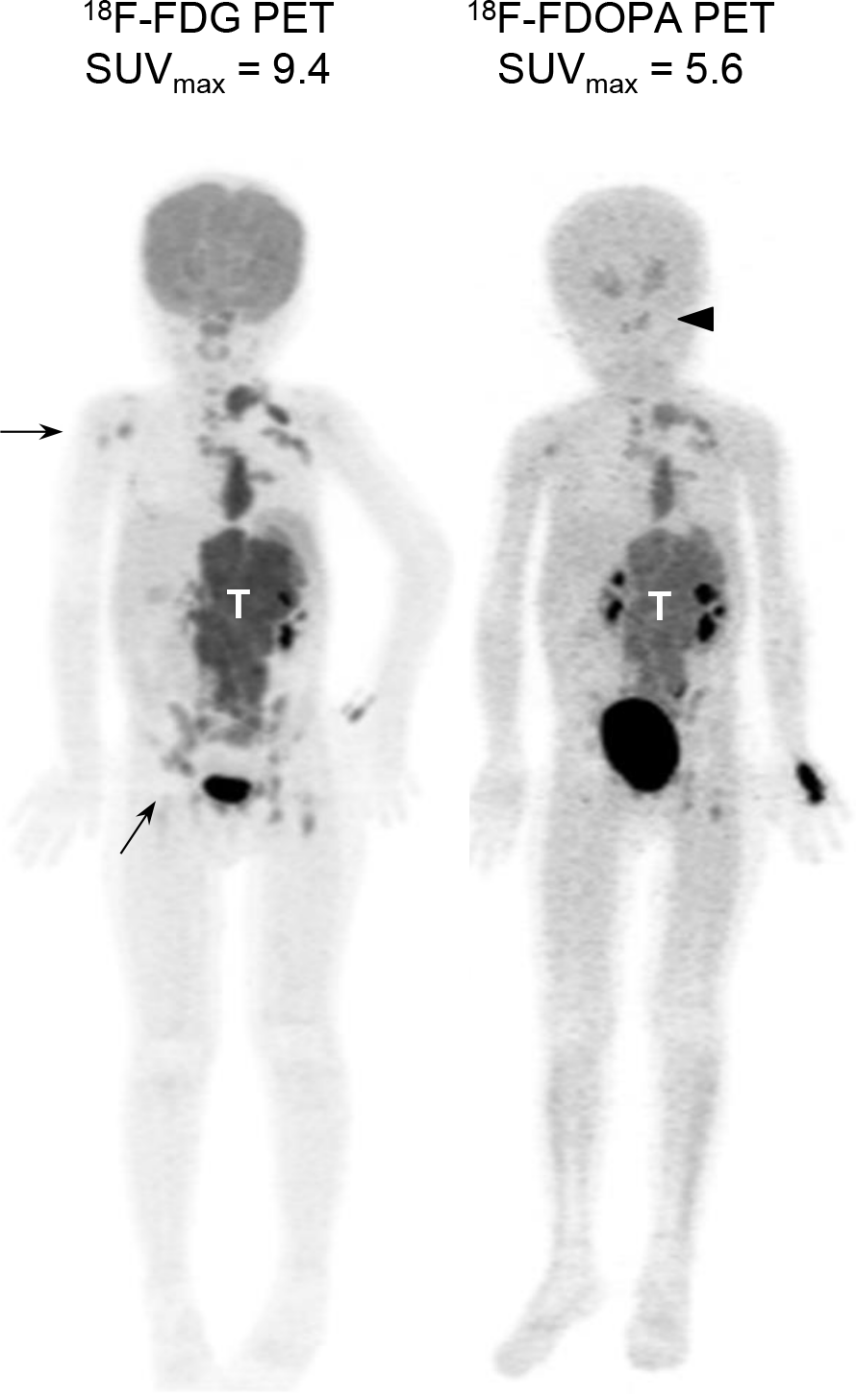
Diagnostic PET imaging with FDG and FDOPA Representative maximum intensity projection images of FDG and FDOPA PET scans in a 4-year-old girl with stage 4, *MYCN*-amplified neuroblatoma at diagnosis. Both scans identified the main tumor (T) with multiple metastases. FDG PET detected more bony lesions in the right humerus and pelvis (arrows), while FDOPA PET provided better contrast to define skull base lesions (arrowhead).

Whole-body PET scans using FDG or FDOPA provided good spatial resolution and clear contrast in the bony compartments and complement each other (Figure 1). Although the strong physiologic FDG uptake by the brain and nasopharynx interfered the interpretation of skull lesions, FDOPA PET helped to identify lesions in the head and neck region more accurately. These imaging features are consistent with our previous findings [18].

### FDG and FDOPA uptake by primary tumors and their clinical characteristics

Reading the FDG and FDOPA PET images of NB, we noted that primary NB tumors frequently took up FDG and FDOPA at different intensity. In this study, the maximum standardized uptake value (SUV_max_) of each primary tumor was measured as the indicator of tumor uptake of FDG or FDOPA. The SUV_max_ was used because of its lower sensitivity to partial-volume effects and higher reproducibility between observers.

Table 1 shows the tumor uptake values compared by clinical characteristics. The SUV_max_ of FDG and the ratio between FDG and FDOPA uptake (G:D) were significantly higher in patients with high-risk features, including older age, stage 4, *MYCN* amplification, and anatomical image-defined risk factors (IDRFs) [5] (all *P* < 0.05). By contrast, the SUV_max_ of FDOPA was significantly higher in patients with younger age, lower stage, and low- or intermediate-risk groups (all *P* < 0.02).

**Figure 2:**
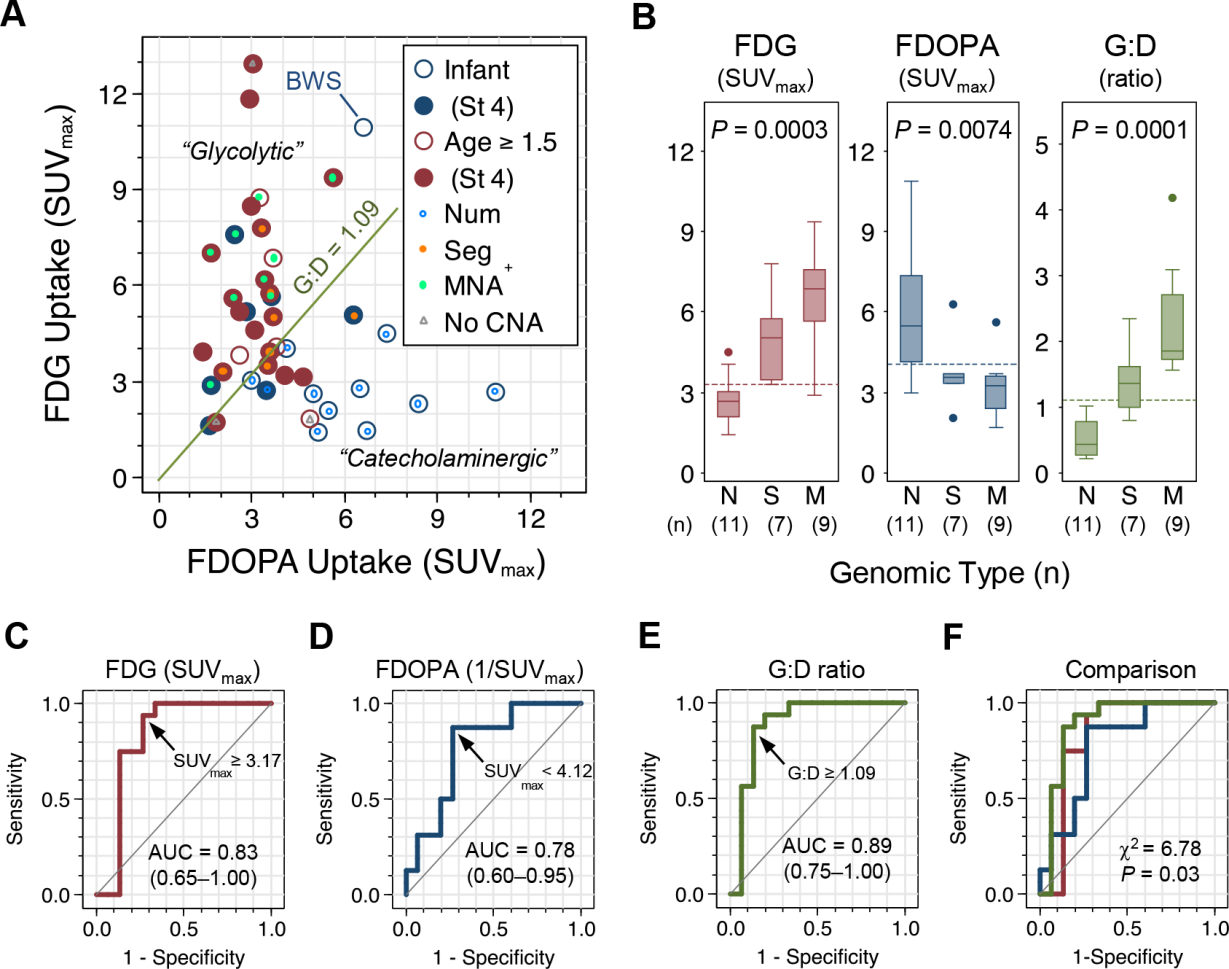
FDG and FDOPA uptake by primary NB tumors and their association with genomic types (**A**) Scatter plot of FDG and FDOPA uptake by primary NB tumors (*n* = 42) with clinical characteristics annotated. (**B**) The three major genomic types of NB showed distinctive FDG and FDOPA uptake patterns. Dashed lines indicate the cutoff value identified by ROC analysis. (**C–F**) ROC analysis of tumor uptake in classifying the poor-risk Seg^+^ and MNA^+^ genomic types. Abbreviations: AUC = area under curve; BWS, Beckwith–Wiedemann syndrome with *IGF2* microdeletion; CNA = copy number alterations; MNA^+^ = M = MYCN amplification; Num^+^ = N = numerical chromosomal alterations; ROC, receiver operating characteristics; Seg^+^ = S = segmental chromosomal alterations; St 4 = stage 4.

The distribution of FDG, FDOPA, or G:D values is skewed with a right tail (Supplementary Figure S2A). On scatter plot, the FDG and FDOPA uptake follows an “L”-shaped distribution (Figure 2A and Supplementary Figure S2B; Spearman's ρ = −0.33, *P* = 0.22) which can be divided into two clusters by the G:D value that distinguishes the major genomic types of NB (presented below): (1) A “glycolytic” group (G:D ≥ 1.09; *n* = 23) featured by higher FDG (FDG^hi^) and lower FDOPA (FDOPA^lo^) uptake; and (2) a “catecholaminergic” group (G:D < 1.09; *n* = 19) with lower FDG (FDG^lo^) and higher FDOPA (FDOPA^hi^) uptake. Tumors with high-risk features, *i.e.* older age (18/23 *vs*. 6/19; *P* = 0.004), stage 3/4 disease (23/23 *vs*. 11/19; *P* = 0.001), and *MYCN* amplification (9/23 *vs*. 0/19; *P* = 0.002), are enriched in the “glycolytic” group. By contrast, tumors from infants diagnosed at < 18 months of age (13/19 vs. 5/23; *P* = 0.004), lower stage patients (8/19 vs. 0/23 with stage 1/2/4S; *P* = 0.001), and *MYCN*-non-amplified patients (19/19 vs. 14/23; *P* = 0.002), were enriched the “catecholaminergic” group.

### Tumor uptake distinguishes NB genomic types

In the study cohort, 31 of 42 patients had their genomic type of NB determined by array-based comparative genomic hybridization (array-CGH) and/or chromogenic *in situ* hybridization. Twenty-seven (87%) out of the 31 patients with available genomic data showed copy number alterations that fall into one of the three major genomic types in NB [22]: Numerical (whole-chromosomal) alterations (Num^+^; *n* = 11), segmental chromosomal alterations (Seg^+^; *n* = 7), or *MYCN*-amplification (MNA^+^; *n* = 9). In the other four patients with genomic data available, three had no significant copy number alterations (*i.e.* silent or “flat”) and one had Beckwith–Wiedemann syndrome with IGF2 microdeletion.

Comparing tumor uptake of FDG and FDOPA by the three major genomic types, FDG uptake of the primary tumor is highest in MNA^+^, intermediate in Seg^+^, and lowest in Num^+^. By contrast, FDOPA uptake is highest in Num^+^ and lowest in Seg^+^ and MNA^+^ (Figure 2B and Table 1). Receiver operating characteristics (ROC) analysis identified that FDG^hi^ with SUV_max_≥ 3.31, FDOPA^lo^ with SUV_max_ < 4.12, and G:D ratio ≥ 1.09 are the cutoff values that best distinguish the unfavorable Seg^+^ and MNA^+^ genomic types from the favorable Num^+^ and other genomic type of NB (Figure 2C–2E). Comparing the area under ROC curves (AUC) of FDG uptake, inversion of FDOPA uptake (1/SUV_max_), and G:D ratio in classifying the unfavorable genomic types of NB (Seg^+^ and MNA^+^), G:D showed a significantly better performance than FDG or FDOPA (*P* = 0.03; Figure 2F) with a sensitivity of 87.5% (61.7%–98.4%) and specificity of 86.7% (59.5%–98.3%). Combining the criteria of FDG SUV_max_ ≥ 3.31 and FDOPA SUV_max_ < 4.12 to define an ultra-high-risk group by PET (PET-UHR; Supplementary Figure S2B) yields a sensitivity of 81.3% (54.4%–96.0%) and specificity of 93.3% (68.1%–99.8%) with an estimated AUC of 0.87 in predicting Seg^+^ or MNA^+^ genomic types of NB (Table 2).

**Table 2:** Diagnostic power of tumor uptake parameters in predicting the unfavorable genomic types* of neuroblastoma

Statistics (95% CI)	FDG Uptake (SUV_max_ ≥ 3.31)	FDOPA Uptake (SUV_max_ < 4.12)	G:D Ratio (≥ 1.09)	PET-UHR*
TP/FN FP/TN	15/1 4/11	14/2 4/11	14/2 2/13	13/3 1/14
Sensitivity	93.8% (69.8%–99.8%)	87.5% (61.7%–98.4%)	87.5% (61.7%–98.4%)	81.3% (54.4%–96.0%)
Specificity	73.3% (44.9%–92.2%)	73.3% (44.9%–92.2%)	86.7% (59.5%–98.3%)	93.3% (68.1%–99.8%)
ROC area	0.83^‡^ (0.65–1.00)	0.78^‡^ (0.60–0.95)	0.89^‡^ (0.75–1.00)	0.87^§^ (0.76–0.99)
Positive Predictive Value	78.9% (54.4%–93.9%)	77.8% (52.4%–93.6)	87.5% (61.7%–98.4%)	92.9% (66.1%–99.8%)
Negative Predictive Value	91.7% (61.5%–99.8%)	84.6% (54.6%–98.1%)	86.7% (59.5%–98.3%)	82.4% (56.6%–96.2%)

* Including segmental chromosomal alterations (*n* = 7) and *MYCN* amplification (*n* = 9) among 31 patients with genomic data.

†Defined by FDG SUV_max_ ≥ 3.31 and FDOPA SUV_max_ < 4.12.

‡ The areas under ROC curves of FDG, FDOPA, and G:D are significantly different (*χ* = 6.78; *P* = 0.03).

§ Estimated by (Sensitivity + Specificity) / 2.

Abbreviations: TP = true positive; TN = true negative; FP = false positive; FN = false negative; ROC = receiver operating characteristic.

### Prognostic value of FDG and FDOPA uptake by the primary tumors

At a median follow-up of 39.2 months (range, 8.8–100.3 months), the 42 patients have a 5-year event-free survival (EFS) rate of 41% (25%–57%) and a 5-year overall survival (OS) rate of 50% (28%–68%). Patients with older age, stage 4 disease, *MYCN* amplification, high-risk disease, and unfavorable genomic types (Seg^+^ and MNA^+^) had significantly worse EFS and OS rates. By contrast, neither IDRFs nor GTR had an impact on survival (Supplementary Figure S3).

**Figure 3:**
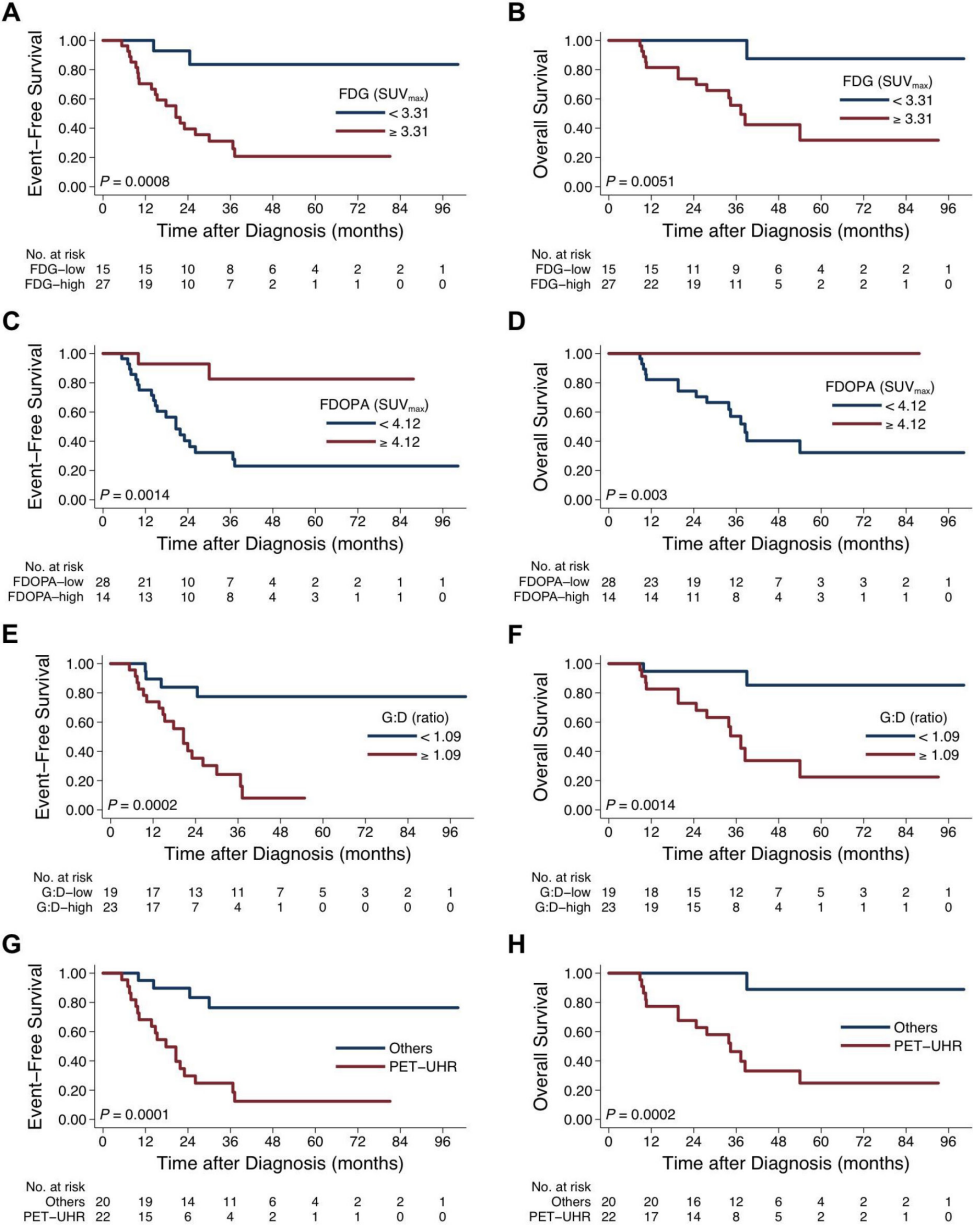
Treatment outcome between patients with different tumor uptake intensity on FDG and FDOPA PET scans Survival curves were compared by FDG uptake (EFS, (**A**) and OS, (**B**), FDOPA uptake (EFS, (**C**) and OS, (**D**), G:D ratio (EFS, (**E**) and OS, (**F**), and PET-defined ultra-high-risk (PET-UHR, FDG SUV_max_ ≥ 3.31 and FDOPA SUV_max_ < 4.12; EFS, (**G**) and OS, (**H**).

Comparing by the values of tumor uptake, strikingly distinct outcomes were found (Figure 3). Specifically, tumors showing higher FDG avidity (SUV_max_ ≥ 3.31; Figure 3A and 3B), lower FDOPA avidity (SUV_max_ < 4.12; Figure 3C and 3D), or higher G:D ratio (ratio ≥ 1.09; Figure 3E and 3F) were associated with significantly worse survival rates (all *P* < 0.01).

Patients of the PET-UHR group (*n* = 22), whose primary tumor showed FDG^hi^ (SUV_max_ ≥ 3.31) and FDOPA^lo^ (SUV_max_ < 4.12) uptake, had extremely poor 5-year EFS (12% [2%–31%] *vs*. 76% [48%–91%]; *P* = 0.0001) and OS (24% [6%–47%] *vs*. 100% [41%–98%]; *P* = 0.0001) rates (Figure 3G and 3H). Among the conventionally high-risk patients as defined by age, stage, histology, and *MYCN* status (*n* = 30) [23, 24], PET-UHR (*n* = 22) showed a trend of worse 5-year EFS (12% [2%–31%] *vs*. 38% [6%–72%]; *P* = 0.16) and OS (24% [6%–47%] *vs*. 50% [1%–91%]; *P* = 0.07).

Cox proportional hazard modeling showed that, on univariate analysis, age, stage, *MYCN* status, FDG^hi^, FDOPA^lo^, G:D **≥** 1.09, and PET-UHR were associated with inferior EFS on univariate analysis (Table 3). On multivariate analysis, PET-UHR was confirmed as a poor prognostic factor (hazard ratio, 4.9 [1.4–16.9]; *P* = 0.012) that was independent from age, stage, IDRF, and *MYCN* status (Table 3). When FDG uptake, FDOPA uptake, or G:D ratio was analyzed with the traditional risk factors respectively, FDG^hi^ or FDOPA^lo^ each predicted inferior EFS independently, while G:D was not significant (Supplementary Table 1).

**Table 3:** Cox proportional hazard modeling of event-free survival

Variable	Univariate			Multivariate		
	HR	95% CI	*P*	HR	95% CI	*P*
Age ≥ 18 months	4.6	1.5–13.7	0.006	0.8	0.2–2.8	0.741
Stage 4	10.1	2.4–45.6	0.002	7.4	1.5–35.4	0.012
*MYCN* amplification	4.0	1.6–10.1	0.004	4.2	1.5–11.9	0.006
IDRF+	2.1	0.8–5.7	0.145	0.6	0.2–1.8	0.382
PET-UHR	6.9	2.3–20.8	0.001	4.9	1.4–16.9	0.012
FDG^hi^ (SUV_max_ ≥ 3.31)	8.1	1.9–35.0	0.005			
FDOPA^lo^ (SUV_max_ < 4.12)	7.5	1.8–32.4	0.007			
Higher G:D ratio (≥ 1.09)	6.3	2.1–19.2	0.001			

Abbreviations: 95%CI = 95% confindence interval; HR = hazard ratio; IDRF+ = presence of image-defined risk factor(s); PET-UHR = ultra-high-risk tumor uptake pattern on FDG and FDOPA PET, defined as FDG SUVmax ≥ 3.31 and FDOPA SUVmax < 4.12.

### Tumor uptake of FDG and FDOPA and gene expression

**Figure 4:**
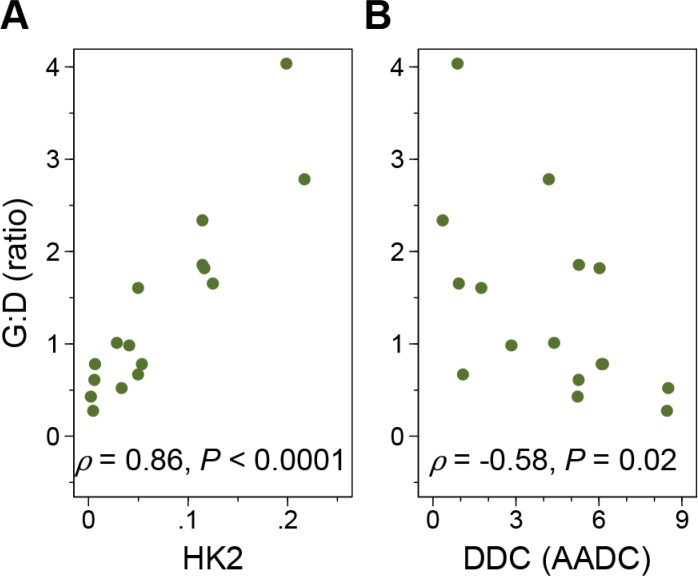
The FDG:FDOPA ratio of tumor uptake and gene expression G:D correlated positively with the hexokinase 2 (*HK2*) (**A**) and negatively with DOPA decarboxylase (*DDC*) (**B**) expression levels. The *x* axis represents the relative folds of target gene expression normalized to the geometric mean of *HPRT1* and *SDHA* transcript levels serving as controls.

To evaluate the probable mechanisms of tumor uptake, we analyzed the expression of selected PET imaging-related genes in 16 primary tumor samples (Figure 4 and Supplementary Figure S4). G:D correlated strongly with hexokinase 2 (*HK2*) expression (Spearman's ρ = 0.86, *P* < 0.0001) and negatively with *DDC* expression (ρ = −0.58, *P* = 0.02). Although hexokinase 1 (*HK1*) also phosphorylates FDG and is expressed at a much higher levels, there was no correlation between *HK1* expression and tumor uptake. Surprisingly, FDOPA uptake had no significant correlation with *TH* (encoding tyrosine hydroxylase upstream of AADC; *P* = 0.09) or *DDC* (*P* = 0.34) expression levels, but showed positive correlation with the expression of *SLC6A2*, the target of ^123^I-MIBG scintigraphy (ρ = 0.68, *P* = 0.004), supporting our previous finding that ^123^I-MIBG avidity was associated with higher FDOPA uptake [18].

## DISCUSSION

The background of this study is the lack of commercially available ^123^I-MIBG in Taiwan during the study period, which has driven us to explore the clinical use of FDG and FDOPA PET in NB. Although we have demonstrated a complementary role of these PET scans in the diagnosis of NB, we must stress that ^123^I-MIBG scan, in countries with a stable supply, remains the single most important molecular imaging of NB. The more recent application of PET with ^124^I-MIBG [25] or ^18^F-labeled MIBG analogs [26, 27] may further enhance the diagnostic power of the “MIBG family” of molecular imaging techniques.

To the best of our knowledge, this is the first study showing significant prognostic impact of both FDG and FDOPA PET at initial diagnosis of NB. We showed that the molecular imaging phenotype of FDG^hi^, FDOPA^lo^ is associated with unfavorable clinico-genomic features and worse treatment outcome in NB. The clinical implications of our findings are two-fold.

First, FDG^hi^, FDOPA^lo^ is associated with unfavorable clinical characteristics but also with the poor-risk Seg^+^ and MNA^+^ genomic types, indicating a strong relationship between metabolic patterns on PET and underlying tumor biology. Although data on age, stage, histopathology, and *MYCN* amplification can be confidently obtained in most clinical settings [24], the detection of Seg^+^, that accounts for 37% of NB and confers a dismal outcome [3], requires time- and resource-consuming pangenomic methods or multiple *in situ* hybridizations [22]. The proposed “PET-UHR” criteria yielded a positive predictive value of 92.9% in predicting Seg^+^ or MNA^+^, providing a convenient parameter of the underlying tumor biology for patients undergoing diagnostic PET scans. Furthermore, area under the ROC curve of G:D is larger than that of FDG or FDOPA uptake alone in classifying the unfavorable genomic types, indicating that combining the information of FDG and FDOPA uptake may serve as a useful biomarker to predict the underlying tumor biology in NB.

Interestingly, while the FDG SUV_max_ was sequentially higher in the three genomic types (Num^+^ < Seg^+^ < MNA^+^), the FDOPA SUV_max_ is equally lower in the two unfavorable types (Num^+^ > Seg^+^ = MNA^+^). Since both the Seg^+^ and MNA^+^ genomic types are associated with the FDG^hi^, FDOPA^lo^ phenotype and a very poor outcome [3], the MYCN oncoprotein itself or its downstream signals may further contribute to a metabolic phenotype towards hyper-glycolysis [28], causing significantly higher FDG uptake and more aggressive behavior of MNA^+^ tumors.

The second clinical implication of our study is that the signal intensity of tumor uptake, *i.e.* FDG^hi^ and/or FDOPA^lo^, serves as independent predictors of inferior prognosis. Based on ^123^I-MIBG scintigraphy, semi-quantitative scoring systems have been developed to assess the total extent of MIBG-avid lesions and predicted the treatment outcome in NB [9, 10]. Adopting these scoring systems of tumor extensity to FDG [14] and FDOPA PET [21] was also prognostic in relapsed/refractory NB. In addition to assessing disease extensiveness, measuring the intensity of tumor uptake may further expand the usefulness of PET scans in NB.

On FDG PET, higher SUV_max_ correlated with higher stage at diagnosis [15, 29] and worse survival at relapse [14]. Interestingly, the FDG uptake values in this study cohort are relatively similar to the results from relevant subgroups in two recent Korean studies (Supplementary Table 2) [15, 29], although the measurement of SUV_max_ may vary with different instruments and imaging protocols. Appropriate instrumentation calibration, standardization of protocols for patient preparation and scanning, and stricter uptake-time control with effective correction algorithms [30] may eventually enable FDG SUV_max_ to become a reproducible imaging biomarker in multi-center and multi-national trials of NB.

On FDOPA PET, Piccardo *et al.* showed that higher “whole-body metabolic burden”, defined as the sum of bony metastatic extent plus the product of tumor uptake and tumor volume, correlated with very poor outcome in relapsed/refractory NB; however, the prognostic impact of the mean or maximal uptake of FDOPA by the primary tumor was not reported [21]. More studies are needed to further delineate the impact of FDG and FDOPA uptake across patient populations.

The biological basis of FDG^hi^, FDOPA^lo^ may largely be explained by the Warburg effect [16]. Both FDG^hi^ and FDOPA^lo^ phenotypes correlated with higher *HK2* expression, which is required for oncogenic transformation *in vitro* and tumor initiation *in vivo* [31] and is enhanced by MYCN and HIF-1α [32]. In breast cancer, the gene expression profile of tumors with FDG SUV_max_ > 10 showed an “FDG signature” that was enriched with glycolysis-related genes and associated with activation of the *MYC* transcription factor (c-Myc), a functional counterpart of *MYCN* [33]. In this study, we demonstrated that NB with *MYCN* amplification correlated with higher FDG uptake.

In addition to its prognostic impact, the glycolysis pathway *per se* may serve as a therapeutic target. It has been recently found that treating NB cells and mouse models with 2-deoxyglucose, an analog of FDG and inhibitor of glycolysis, decreased *HK2* expression and induced apoptosis [34]. Preclinical testing has also proved the *in vivo* efficacy of 2-deoxyglucose in NB [35]. Based on these findings, PET imaging may be further studied as a biomarker that identifies patients with NB or other cancers who would most likely to benefit from novel therapies targeting *MYC/MYCN* [28, 36] or the glycolytic pathway [34, 35, 37].

Surprisingly, we found that lower, rather than higher, FDOPA uptake significantly correlated with very poor prognosis. The high uptake of FDOPA in NB and its association with better outcome may be explained by its characteristic catecholaminergic differentiation. Although FDOPA uptake did not correlate with the degree of differentiation on histopathology, its negative correlation with *HK2* and positive correlation with *SLC6A2* expression levels suggests that FDOPA^lo^ tumors may confer a hyper-glycolytic and catecholaminergically dedifferentiated phenotype at the molecular level. Characterization of an “FDOPA signature” of gene expression is warranted.

Our study has some limitations. As the tumor uptake values are continuous variables, they may have continuous impact on prognosis, similar to the age effect in NB [38]. Our sample size precluded extensive statistical analyses to search for the best cutoffs that predict the highest risk of incomplete resection or poor prognosis. We postulate that PET scans may add value to anatomical imaging and conventional risk stratification systems by identifying a subgroup of patients with excellent prognosis, who can truly benefit from modern multimodal therapy, from the ultra-high-risk patients who should be enrolled in novel therapeutic trials. However, this observation cannot yet be made with assurance. Our data support that the utility of diagnostic FDG and/or FDOPA PET should be further evaluated in a larger cohort of high-risk NB, ideally through multi-center or international collaborations.

In conclusion, we showed that the tumor uptake intensity of FDG and FDOPA on PET scans at diagnosis distinguish the tumor biology and treatment outcome in NB. The unfavorable PET imaging phenotype with FDG^hi^, FDOPA^lo^ uptake patterns were associated with poor-risk clinico-genomic features and worse prognosis. The prognostic value of PET-UHR, using FDG SUV_max_ ≥ 3.31 and FDOPA SUV_max_ < 4.12 as criteria, may be incorporated in future risk stratification systems and be validated in prospective trials.

## MATERIALS AND METHODS

### Patient enrollment

From June 2007 to July 2014, patients diagnosed with NB were enrolled. FDG and FDOPA PET scans were performed at diagnosis and during follow-up at National Taiwan University Hospital, Taipei, Taiwan at an interval of 3–12 months with other standard evaluations. Patients with paired FDG and FDOPA PET scans performed before completion of the first chemotherapy cycle were included for analysis (Figure 1). The study was approved by National Taiwan University Hospital Research Ethics Committee. Informed consent was obtained from each participant's guardian.

### Standard evaluations for neuroblastoma patients

In addition to PET imaging, routine evaluations included complete blood count, basic biochemistry, serum lactate dehydrogenase and ferritin levels, urinary vanilylmandelic acid level, CT/MR imaging, and bone marrow smear and biopsy. The presence of CT/MR image-defined risk factor was retrospectively retrieved from patients' radiological reports at diagnosis, based on the International Neuroblastoma Risk Group staging system criteria [5]. All patients were staged according to International Neuroblastoma Staging System [39]. The histological classification of resected tumors was based on the International Neuroblastoma Pathology Classification [40]. *MYCN* amplification was determined by chromogenic *in situ* hybridization [41]. DNA ploidy and urinary homovanillic acid level were not routinely evaluated.

The major genomic type of each patient's tumor, *i.e.* the presence of segmental or numerical chromosomal alterations, was determined by array-CGH [3, 4, 22] using BAC-based (CMDX, Irvine, CA; resolution, 1 Mb) or CytoChip Oligo (BluGnome, Cambridge, UK; resolution, 60 kb) platforms.

### Risk-Directed therapy

Patients were treated and followed according to Taiwan Pediatric Oncology Group's TPOG-N2002 [23, 42], a nationwide, risk-directed protocol for NB with modification of the Children's Oncology Group (COG)'s regimens [43, 44]. The complete design and results of TPOG-N2002 are to be presented elsewhere (Dr. Rong-Long Chen *et al.*, manuscript in preparation). Briefly, patients were stratified into three risk groups based on age, stage, *MYCN*, and histology using a modified Children's Oncology Group (COG) definition [24] without ploidy. Briefly, the low-risk group includes all stage 1 patients, all stage 2 infants and children with normal *MYCN* copy number, stage 2 children with *MYCN* amplification and favorable histology, and stage 4S with normal *MYCN* and favorable histology. The intermediate-risk group includes stage 3 or 4 infants; stage 3 children with favorable histology; and stage 4S infants with unfavorable histology and normal *MYCN*. The high-risk group includes stage 2 with *MYCN* amplification and unfavorable histology, stage 3 with *MYCN* amplification, stage 3 children with unfavorable histology, stage 4 patients who are older than 365 days or have *MYCN* amplification, and stage 4S with *MYCN* amplification [23].

According to their risk grouping [23, 24], patients were treated with surgery alone; surgery plus 4 or 8 chemotherapy cycles, as modified from COG-A3961 [43]; or multimodal therapy including surgery, chemotherapy, autologous stem cell transplantation, radiotherapy, and 13-*cis*-reticnoic acid, as modified from COG-A3973 [44].

### Acquisition of PET images

FDG and FDOPA were produced with commercial systems (TRACERlab, GE Healthcare, USA) [18, 45]. FDG and FDOPA PET scans were performed separately, at least one day apart. Before FDG PET, patients were fasted with avoidance of glucose-containing intravenous fluids for 6 hours. Before FDOPA PET, patients received 2 mg/kg of carbidopa orally 1 hour before injection [46]. Forty-five minutes after FDG (5 MBq/kg) or 90 minutes after FDOPA (4 MBq/kg) injection, whole-body images were acquired on a PET/CT scanner with low-dose CT (Discovery ST-16, GE Medical Systems, Milwaukee, WI). We began sedation 30 minutes before scanning if necessary.

Images were reconstructed using an iterative algorithm provided by the machine manufacturer. Analysis was performed on attenuation-corrected images. The maximum standardized uptake value (SUV_max_) was determined by manually drawing elliptical regions of interest around areas of abnormal uptake, using the software from GE Medical System on a Xeleris 2^®^ workstation (GE Healthcare). For patients with two or more primary tumors (*n* = 3), tumor with the largest volume was analyzed.

### Quantitative real-time PCR

Total RNA was extracted from frozen tumor tissue with Trizol reagent following the manufacturer's instructions (Invitrogen). cDNA was produced from 5 mg of RNA using Superscript III with random hexamer primers (Invitrogen). Analysis of gene expression was performed in Applied Biosystems 7500 Fast Real-Time PCR System by using specific TaqMan primers (Applied Biosystems) for *HK1* (Hs00175976_m1), *HK2* (Hs00606086_m1), *TH* (Hs00165941_m1), *DDC* (Hs01105048_m1), *SLC6A2* (Hs00426573_m1), *HPRT1* (Hs99999909_m1), and *SDHA* (Hs00188166_m1). Expression levels of PET-related genes were averaged from two replicates and normalized to the geometric mean of *HPRT1* and *SDHA*, which are control genes with the least expression variability across NB samples [47].

### Statistical analysis

Outcome data were frozen on October 9, 2015. The Fisher exact, Kruskal-Wallis, and Spearman's nonparametric correlation tests were used to evaluate the association across variables. The cutoff value of PET imaging parameters was determined by ROC curve analysis. EFS was calculated from diagnosis to the first occurrence of relapse, progression, secondary malignancy, or death, or to the last contact if no event occurred. OS was calculated until the time of death or until the last contact that the patient was alive. Kaplan-Meier curves were generated and compared by log-rank tests. Cox proportional hazard models were built to test for prognostic values. The statistical analyses were performed with Small Stata 11.0 software (StataCorp, College Station, TX). All tests were two-sided. *P* values < 0.05 were considered statistically significant.

## SUPPLEMENTARY MATERIALS FIGURES AND TABLES



## References

[1] Maris JM (2010). Recent advances in neuroblastoma. N Engl J Med.

[2] Kushner BH (2004). Neuroblastoma: a disease requiring a multitude of imaging studies. J Nucl Med.

[3] Janoueix-Lerosey I, Schleiermacher G, Michels E, Mosseri V, Ribeiro A, Lequin D, Vermeulen J, Couturier J, Peuchmaur M, Valent A, Plantaz D, Rubie H, Valteau-Couanet D (2009). Overall genomic pattern is a predictor of outcome in neuroblastoma. J Clin Oncol.

[4] Tomioka N, Oba S, Ohira M, Misra A, Fridlyand J, Ishii S, Nakamura Y, Isogai E, Hirata T, Yoshida Y, Todo S, Kaneko Y, Albertson DG (2008). Novel risk stratification of patients with neuroblastoma by genomic signature, which is independent of molecular signature. Oncogene.

[5] Monclair T, Brodeur GM, Ambros PF, Brisse HJ, Cecchetto G, Holmes K, Kaneko M, London WB, Matthay KK, Nuchtern JG, von Schweinitz D, Simon T, Cohn SL (2009). The International Neuroblastoma Risk Group (INRG) staging system: an INRG Task Force report. J Clin Oncol.

[6] Brisse HJ, McCarville MB, Granata C, Krug KB, Wootton-Gorges SL, Kanegawa K, Giammarile F, Schmidt M, Shulkin BL, Matthay KK, Lewington VJ, Sarnacki S, Hero B (2011). Guidelines for imaging and staging of neuroblastic tumors: consensus report from the International Neuroblastoma Risk Group Project. Radiology.

[7] Kushner BH, Kramer K, Modak S, Cheung NK (2009). Sensitivity of surveillance studies for detecting asymptomatic and unsuspected relapse of high-risk neuroblastoma. J Clin Oncol.

[8] Dubois SG, Geier E, Batra V, Yee SW, Neuhaus J, Segal M, Martinez D, Pawel B, Yanik G, Naranjo A, London WB, Kreissman S, Baker D (2012). Evaluation of Norepinephrine Transporter Expression and Metaiodobenzylguanidine Avidity in Neuroblastoma: A Report from the Children's Oncology Group. Int J Mol Imag.

[9] Yanik GA, Parisi MT, Shulkin BL, Naranjo A, Kreissman SG, London WB, Villablanca JG, Maris JM, Park JR, Cohn SL, McGrady P, Matthay KK (2013). Semiquantitative mIBG Scoring as a Prognostic Indicator in Patients with Stage 4 Neuroblastoma: A Report from the Children's Oncology Group. J Nucl Med.

[10] Decarolis B, Schneider C, Hero B, Simon T, Volland R, Roels F, Dietlein M, Berthold F, Schmidt M (2013). Iodine-123 metaiodobenzylguanidine scintigraphy scoring allows prediction of outcome in patients with stage 4 neuroblastoma: results of the cologne interscore comparison study. J Clin Oncol.

[11] Wilson JS, Gains JE, Moroz V, Wheatley K, Gaze MN (2014). A systematic review of 131I-meta iodobenzylguanidine molecular radiotherapy for neuroblastoma. Eur J Cancer.

[12] Shulkin BL, Hutchinson RJ, Castle VP, Yanik GA, Shapiro B, Sisson JC (1996). Neuroblastoma: positron emission tomography with 2-[fluorine-18]-fluoro-2-deoxy-D-glucose compared with metaiodobenzylguanidine scintigraphy. Radiology.

[13] Sharp SE, Shulkin BL, Gelfand MJ, Salisbury S, Furman WL (2009). 123I-MIBG scintigraphy and 18F-FDG PET in neuroblastoma. J Nucl Med.

[14] Papathanasiou ND, Gaze MN, Sullivan K, Aldridge M, Waddington W, Almuhaideb A, Bomanji JB (2011). 18F-FDG PET/CT and 123I-Metaiodobenzylguanidine Imaging in High-Risk Neuroblastoma: Diagnostic Comparison and Survival Analysis. J Nucl Med.

[15] Choi YJ, Hwang HS, Kim HJ, Jeong YH, Cho A, Lee JH, Yun M, Lee JD, Kang WJ (2014). (18)F-FDG PET as a single imaging modality in pediatric neuroblastoma: comparison with abdomen CT and bone scintigraphy. Ann Nucl Med.

[16] Koppenol WH, Bounds PL, Dang CV (2011). Otto Warburg's contributions to current concepts of cancer metabolism. Nat Rev Cancer.

[17] Lopci E, Piccardo A, Nanni C, Altrinetti V, Garaventa A, Pession A, Cistaro A, Chiti A, Villavecchia G, Fanti S (2012). 18F-DOPA PET/CT in Neuroblastoma: Comparison of Conventional Imaging With CT/MR. Clin Nucl Med.

[18] Lu MY, Liu YL, Chang HH, Jou ST, Yang YL, Lin KH, Lin DT, Lee YL, Lee H, Wu PY, Luo TY, Shen LH, Huang SF (2013). Characterization of neuroblastic tumors using 18F-FDOPA PET. J Nucl Med.

[19] Piccardo A, Lopci E, Conte M, Garaventa A, Foppiani L, Altrinetti V, Nanni C, Bianchi P, Cistaro A, Sorrentino S, Cabria M, Pession A, Puntoni M (2012). Comparison of (18)F-dopa PET/CT and (123)I-MIBG scintigraphy in stage 3 and 4 neuroblastoma: a pilot study. Eur J Nucl Med Mol Imaging.

[20] Gilbert J, Haber M, Bordow SB, Marshall GM, Norris MD (1999). Use of tumor-specific gene expression for the differential diagnosis of neuroblastoma from other pediatric small round-cell malignancies. Am J Pathol.

[21] Piccardo A, Puntoni M, Lopci E, Conte M, Foppiani L, Sorrentino S, Morana G, Naseri M, Cistaro A, Villavecchia G, Fanti S, Garaventa A (2014). Prognostic value of 18F-DOPA PET/CT at the time of recurrence in patients affected by neuroblastoma. Eur J Nucl Med Mol Imaging.

[22] Schleiermacher G, Mosseri V, London WB, Maris JM, Brodeur GM, Attiyeh E, Haber M, Khan J, Nakagawara A, Speleman F, Noguera R, Tonini GP, Fischer M (2012). Segmental chromosomal alterations have prognostic impact in neuroblastoma: a report from the INRG project. Br J Cancer.

[23] Liu YL, Miser JS, Hsu WM (2014). Risk-directed therapy and research in neuroblastoma. J Formos Med Assoc.

[24] Henderson TO, Bhatia S, Pinto N, London WB, McGrady P, Crotty C, Sun CL, Cohn SL (2011). Racial and ethnic disparities in risk and survival in children with neuroblastoma: a Children's Oncology Group study. J Clin Oncol.

[25] Cistaro A, Quartuccio N, Caobelli F, Piccardo A, Paratore R, Coppolino P, Sperandeo A, Arnone G, Ficola U (2015). 124I-MIBG: a new promising positron-emitting radiopharmaceutical for the evaluation of neuroblastoma. Nucl Med Rev Cent East Eur.

[26] Zhang H, Huang R, Cheung NK, Guo H, Zanzonico PB, Thaler HT, Lewis JS, Blasberg RG (2014). Imaging the norepinephrine transporter in neuroblastoma: a comparison of [18F]-MFBG and 123I-MIBG. Clin Cancer Res.

[27] Vaidyanathan G, Affleck DJ, Zalutsky MR (1994). (4-[18F]fluoro-3-iodobenzyl)guanidine, a potential MIBG analogue for positron emission tomography. J Med Chem.

[28] Zirath H, Frenzel A, Oliynyk G, Segerstrom L, Westermark UK, Larsson K, Munksgaard Persson M, Hultenby K, Lehtio J, Einvik C, Pahlman S, Kogner P, Jakobsson PJ (2013). MYC inhibition induces metabolic changes leading to accumulation of lipid droplets in tumor cells. Proc Natl Acad Sci U S A.

[29] Lee JW, Cho A, Yun M, Lee JD, Lyu CJ, Kang WJ (2015). Prognostic value of pretreatment FDG PET in pediatric neuroblastoma. Eur J Radiol.

[30] Kurland BF, Muzi M, Peterson LM, Doot RK, Wangerin KA, Mankoff DA, Linden HM, Kinahan PE (2016). Multicenter Clinical Trials Using 18F-FDG PET to Measure Early Response to Oncologic Therapy: Effects of Injection-to-Acquisition Time Variability on Required Sample Size. J Nucl Med.

[31] Patra KC, Wang Q, Bhaskar PT, Miller L, Wang Z, Wheaton W, Chandel N, Laakso M, Muller WJ, Allen EL, Jha AK, Smolen GA, Clasquin MF (2013). Hexokinase 2 is required for tumor initiation and maintenance and its systemic deletion is therapeutic in mouse models of cancer. Cancer Cell.

[32] Qing G, Skuli N, Mayes PA, Pawel B, Martinez D, Maris JM, Simon MC (2010). Combinatorial regulation of neuroblastoma tumor progression by N-Myc and hypoxia inducible factor HIF-1alpha. Cancer Res.

[33] Palaskas N, Larson SM, Schultz N, Komisopoulou E, Wong J, Rohle D, Campos C, Yannuzzi N, Osborne JR, Linkov I, Kastenhuber ER, Taschereau R, Plaisier SB (2011). 18F-fluorodeoxy-glucose positron emission tomography marks MYC-overexpressing human basal-like breast cancers. Cancer Res.

[34] Chuang JH, Chou MH, Tai MH, Lin TK, Liou CW, Chen T, Hsu WM, Wang PW (2013). 2-Deoxyglucose treatment complements the cisplatin- or BH3-only mimetic-induced suppression of neuroblastoma cell growth. Int J Biochem Cell Biol.

[35] Huang CC, Wang SY, Lin LL, Wang PW, Chen TY, Hsu WM, Lin TK, Liou CW, Chuang JH (2015). Glycolytic inhibitor 2-deoxyglucose simultaneously targets cancer and endothelial cells to suppress neuroblastoma growth in mice. Dis Model Mech.

[36] Puissant A, Frumm SM, Alexe G, Bassil CF, Qi J, Chanthery YH, Nekritz EA, Zeid R, Gustafson WC, Greninger P, Garnett MJ, McDermott U, Benes CH (2013). Targeting MYCN in neuroblastoma by BET bromodomain inhibition. Cancer Discov.

[37] Bean JF, Qiu YY, Yu S, Clark S, Chu F, Madonna MB (2014). Glycolysis inhibition and its effect in doxorubicin resistance in neuroblastoma. J Pediatr Surg.

[38] London WB, Castleberry RP, Matthay KK, Look AT, Seeger RC, Shimada H, Thorner P, Brodeur G, Maris JM, Reynolds CP, Cohn SL (2005). Evidence for an age cutoff greater than 365 days for neuroblastoma risk group stratification in the Children's Oncology Group. J Clin Oncol.

[39] Brodeur GM, Pritchard J, Berthold F, Carlsen NL, Castel V, Castelberry RP, De Bernardi B, Evans AE, Favrot M, Hedborg F (1993). Revisions of the international criteria for neuroblastoma diagnosis, staging, and response to treatment. J Clin Oncol.

[40] Shimada H, Ambros IM, Dehner LP, Hata J, Joshi VV, Roald B, Stram DO, Gerbing RB, Lukens JN, Matthay KK, Castleberry RP (1999). The International Neuroblastoma Pathology Classification (the Shimada system). Cancer.

[41] Tsai HY, Hsi BL, Hung IJ, Yang CP, Lin JN, Chen JC, Tsai SF, Huang SF (2004). Correlation of MYCN amplification with MCM7 protein expression in neuroblastomas: a chromogenic *in situ* hybridization study in paraffin sections. Hum Pathol.

[42] Chang HH, Lee H, Hu MK, Tsao PN, Juan HF, Huang MC, Shih YY, Wang BJ, Jeng YM, Chang CL, Huang SF, Tsay YG, Hsieh FJ (2010). Notch1 expression predicts an unfavorable prognosis and serves as a therapeutic target of patients with neuroblastoma. Clin Cancer Res.

[43] Baker DL, Schmidt ML, Cohn SL, Maris JM, London WB, Buxton A, Stram D, Castleberry RP, Shimada H, Sandler A, Shamberger RC, Look AT, Reynolds CP (2010). Outcome after reduced chemotherapy for intermediate-risk neuroblastoma. New Engl J Med.

[44] Kreissman SG, Seeger RC, Matthay KK, London WB, Sposto R, Grupp SA, Haas-Kogan DA, Laquaglia MP, Yu AL, Diller L, Buxton A, Park JR, Cohn SL (2013). Purged versus non-purged peripheral blood stem-cell transplantation for high-risk neuroblastoma (COG A3973): a randomised phase 3 trial. Lancet Oncol.

[45] de Vries EFJ, Luurtsema G, Brussermann M, Elsinga PH, Vaalburg W (1999). Fully automated synthesis module for the high yield one-pot preparation of 6-[18F]fluoro-l-DOPA. Appl Radiat Isot.

[46] Timmers HJ, Hadi M, Carrasquillo JA, Chen CC, Martiniova L, Whatley M, Ling A, Eisenhofer G, Adams KT, Pacak K (2007). The effects of carbidopa on uptake of 6-18F-Fluoro-L-DOPA in PET of pheochromocytoma and extraadrenal abdominal paraganglioma. J Nucl Med.

[47] Fischer M, Skowron M, Berthold F (2005). Reliable transcript quantification by real-time reverse transcriptase-polymerase chain reaction in primary neuroblastoma using normalization to averaged expression levels of the control genes HPRT1 and SDHA. J Mol Diagn.

