# Supplementary material for: Diagnostic FDG and FDOPA positron emission tomography scans distinguish the genomic type and treatment outcome of neuroblastoma
Source: Oncotarget. 2016 Mar 5;7(14):18774–86. doi: 10.18632/oncotarget.7933 (PMC4951328; doi:10.18632/oncotarget.7933)
Supplement: Supplementary file 1 [file oncotarget-07-18774-s001.pdf]

Diagnostic FDG and FDOPA positron emission tomography scans distinguish the genomic type and treatment outcome of neuroblastoma

Supplementary Materials

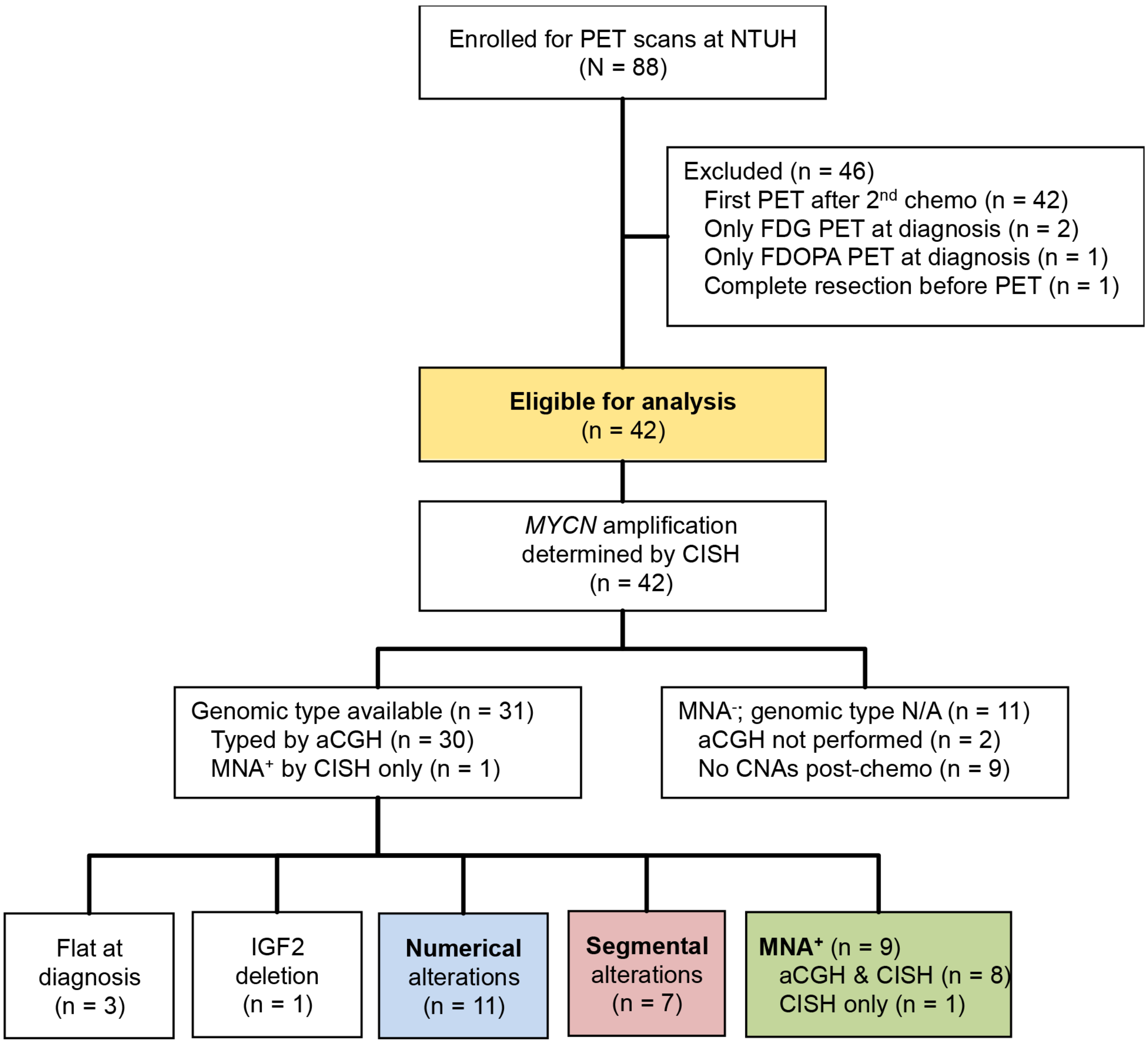

Supplementary Figure S1: Patient flow diagram. Abbreviations: aCGH = array-based comparative genomic hybridization; chemo = chemotherapy; CISH = chromogenic *in situ* hybridization; CR = complete response; MNA = *MYCN* amplification; N/A = not available; NTUH = National Taiwan University Hospital, Taipei, Taiwan.

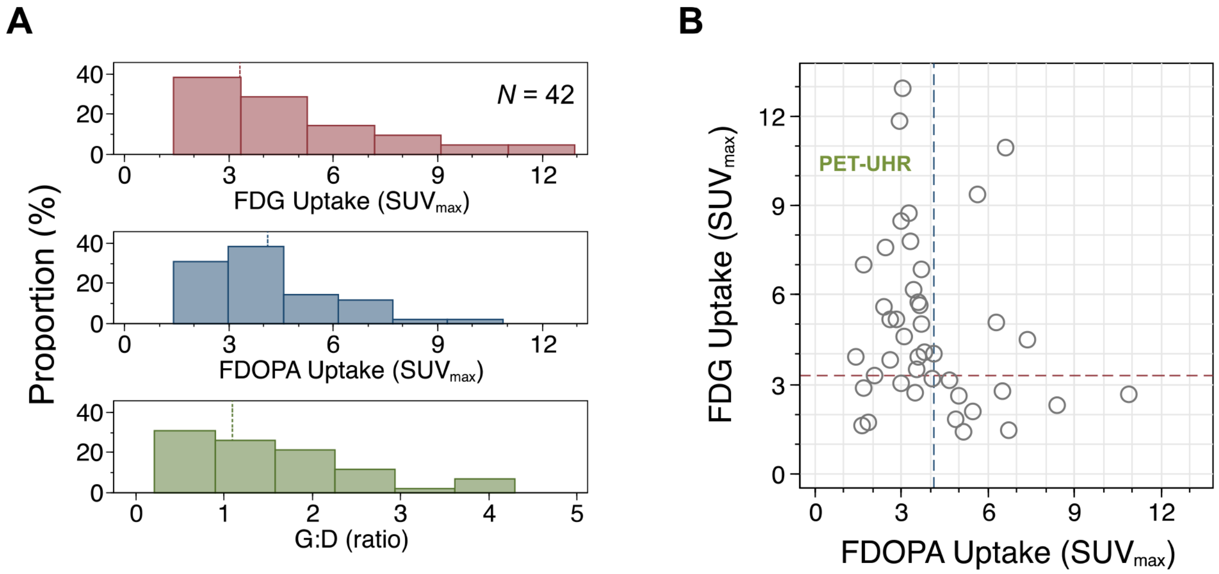

Supplementary Figure S2: FDG and FDOPA uptake by primary NB tumors and their association with genomic types and surgical resection. (**A**) Histograms and (**B**) scatter plot showing the Distribution of FDG uptake, FDOPA uptake, and G:D ratio. Dashed lines indicate the FDG $SUV_{max} \geq 3.31$ and FDOPA SUVmax < 4.12 cutoff identified by receiver operating characteristic analysis. Shaded area indicates PET-UHR. Abbreviations: G:D = ratio between the uptake (SUV_{max}) of primary tumor on FDG and FDOPA PET scans; SUV_{max} = maximum standard uptake value.

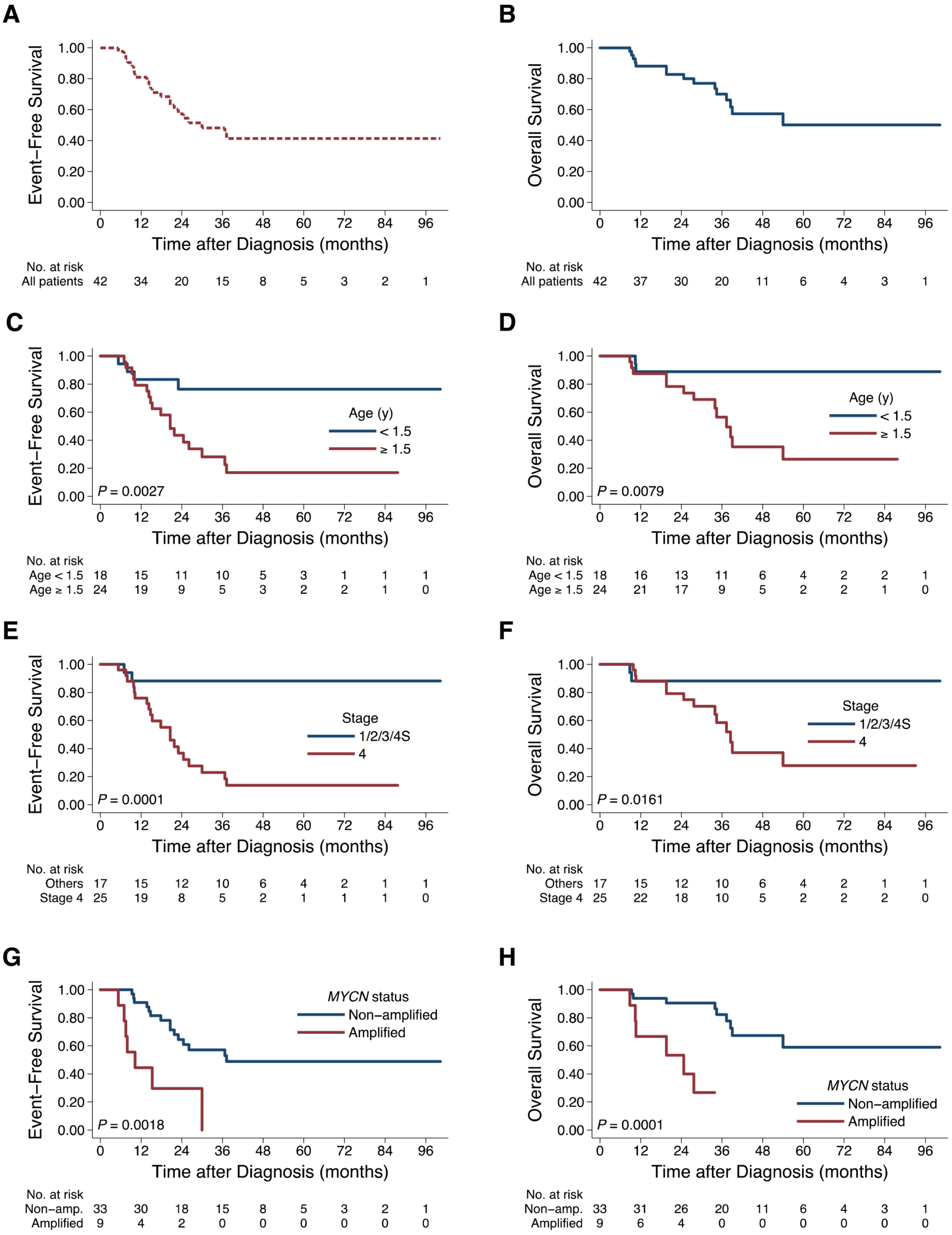
A
Event−Free Survival
Time after Diagnosis (months)
No. at risk
All patients 42 34 20 15 8 5 3 2 1
B
Overall Survival
Time after Diagnosis (months)
No. at risk
All patients 42 37 30 20 11 6 4 3 1
C
Event−Free Survival
Age (y)
< 1.5
≥ 1.5
P = 0.0027
Time after Diagnosis (months)
No. at risk
Age < 1.5 18 15 11 10 5 3 1 1 1
Age ≥ 1.5 24 19 9 5 3 2 2 1 0
D
Overall Survival
Age (y)
< 1.5
≥ 1.5
P = 0.0079
Time after Diagnosis (months)
No. at risk
Age < 1.5 18 16 13 11 6 4 2 2 1
Age ≥ 1.5 24 21 17 9 5 2 2 1 0
E
Event−Free Survival
Stage
1/2/3/4S
4
P = 0.0001
Time after Diagnosis (months)
No. at risk
Others 17 15 12 10 6 4 2 1 1
Stage 4 25 19 8 5 2 1 1 1 0
F
Overall Survival
Stage
1/2/3/4S
4
P = 0.0161
Time after Diagnosis (months)
No. at risk
Others 17 15 12 10 6 4 2 1 1
Stage 4 25 22 18 10 5 2 2 2 0
G
Event−Free Survival
MYCN status
Non−amplified
Amplified
P = 0.0018
Time after Diagnosis (months)
No. at risk
Non−amp. 33 30 18 15 8 5 3 2 1
Amplified 9 4 2 0 0 0 0 0 0
H
Overall Survival
MYCN status
Non−amplified
Amplified
P = 0.0001
Time after Diagnosis (months)
No. at risk
Non−amp. 33 31 26 20 11 6 4 3 1
Amplified 9 6 4 0 0 0 0 0 0

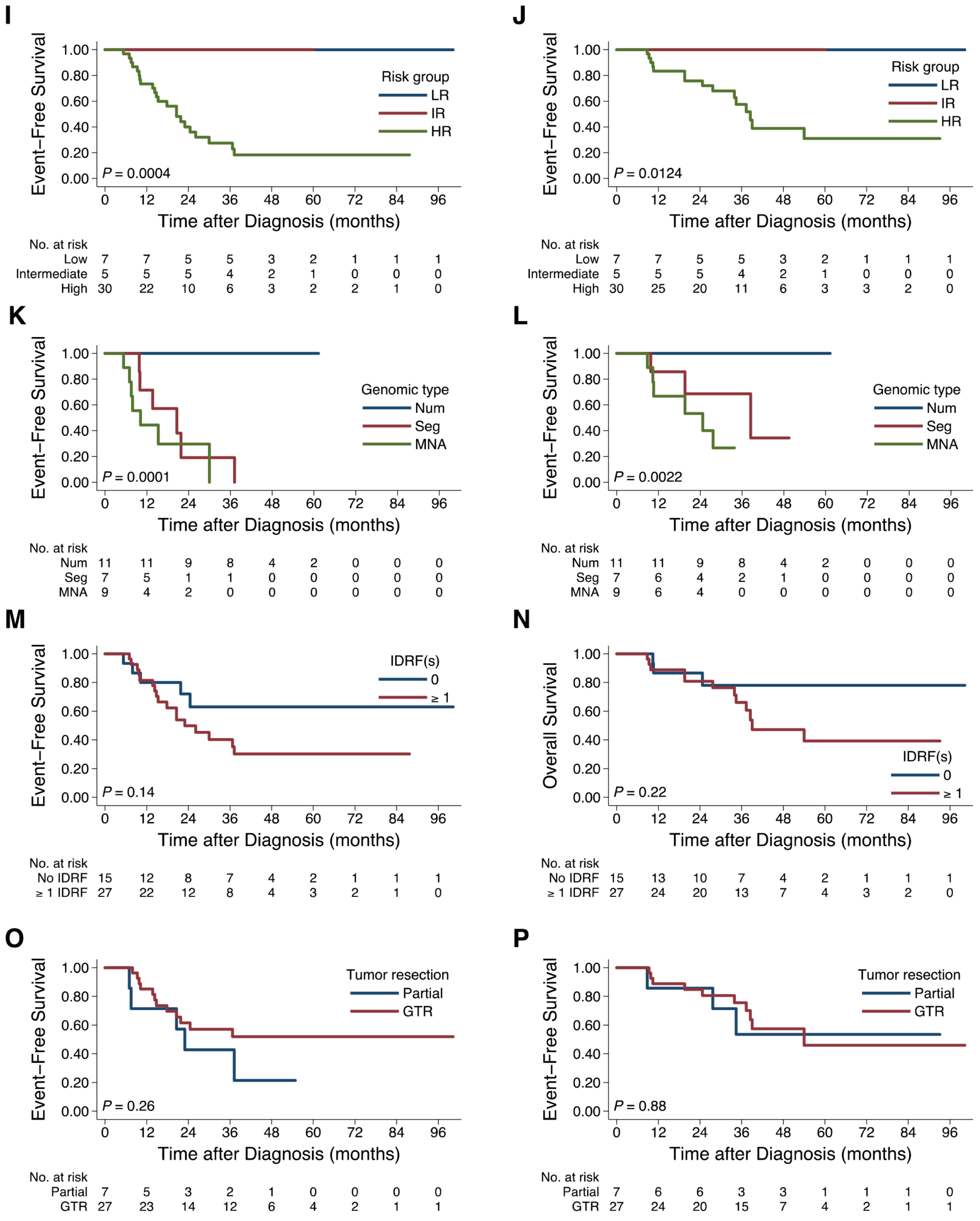

Supplementary Figure S3: Treatment outcome compared by traditional risk risk factors. Survival curves of the study cohort (EFS, (**A**) and OS, (**B**) and compared by age (EFS, (**C**) and OS, (**D**), stage (EFS, (**E**) and OS, (**F**), MYCN amplification (EFS, (**G**) and OS, (**H**), risk group (EFS, (**I**) and OS, (**J**), major genomic type (EFS, (**K**) and OS, (**L**), IDRF (EFS, (**M**) and OS, (**N**), and extent of surgery (EFS, (**O**) and OS, (**P**). Abbreviations: Amp = amplification; GTR = gross total resection; MNA = *MYCN* amplification; Num = numerical chromosomal alterations; Seg = segmemtal chromosomal alterations.

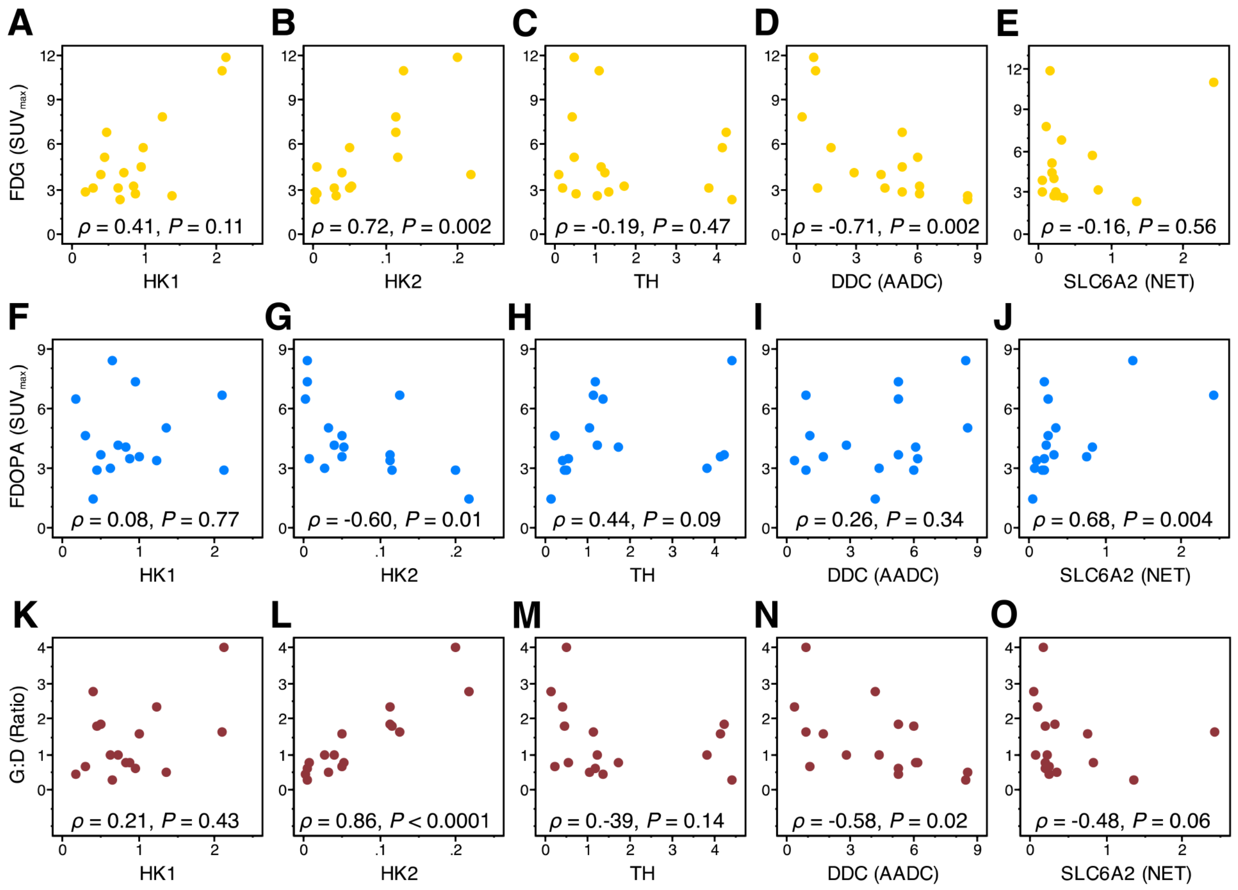

Supplementary Figure S4: Correlation between tumor uptake of FDG (A-E), FDOPA (F-J), or G:D ratio (K-O) and selected gene expression related with glycolysis (*HK1, HK2*) and catecholamine metabolism (*TH, DDC*) and transport (*SLC6A2*). The Spearman's nonparametric correlation coefficient (ρ) and its P value in each correlation test are shown. The x axis represents the relative folds of target gene expression normalized to the geometric mean of HPRT1 and SDHA transcript levels. Abbreviations: G:D = ratio between the SUV_{max} of FDG and FDOPA by the primary tumor; AADC = aromatic l-amino acid decarboxylase; NET = norepinephrine transporter.

Supplementary Table 1: Cox proportional hazard modeling on event-free survival using individual PET imaging parameters

Variable	Multivariate		
	HR	95% CI	*P*
**Model 1: Age, Stage, MYCN, IDRF, and FDG uptake**			
Age ≥ 18 months	0.9	0.2–3.3	0.876
Stage 4	9.3	1.7–51.4	0.011
MYCN amplification	2.5	1.0–6.5	0.058
$IDRF^+$	0.6	0.2–1.6	0.245
FDG^{hi} (SUVmax ≥ 3.31)	5.0	1.0–23.9	0.045
**Model 2: Age, Stage, MYCN, IDRF, and FDOPA uptake**			
Age ≥ 18 months	1.6	0.5–4.9	0.416
Stage 4	6.3	1.4–29.4	0.018
MYCN amplification	4.5	1.6–12.6	0.004
$IDRF^+$	0.7	0.3–2.0	0.534
$FDOPA^{lo}$ (SUVmax < 4.12)	4.8	1.0–22.4	0.047
**Model 3: Age, Stage, MYCN, IDRF, and G:D ratio**			
Age ≥ 18 months	1.3	0.4–4.6	0.673
Stage 4	74	1.3–42.7	0.026
MYCN amplification	2.5	0.9–6.7	0.073
$IDRF^+$	0.6	0.2–1.7	0.311
G:D (ratio ≥ 1.09)	2.0	0.5–7.7	0.290

Abbreviations: 95% CI = 95% confidence interval; HR = hazard ratio; $IDRF^+$ = presence of image-defined risk factor(s); PET-UHR = ultra-high-risk tumor uptake pattern on FDG and FDOPA PET, defined as FDG SUV_{max} ≥ 3.31 and FDOPA SUVmax < 4.12.

Supplementary Table 2: Comparison of FDG uptake by primary neuroblastoma across studies

Study	Subgroup	n	SUVmax*	Equipment	FDG Dose	Uptake Time†
Choi *et al.* [15]	Stage 3/4<br>Stage 1/2	23<br>7	5.45 (1.23–13.72)<br>3.03 (0.94–7.49)	GE Advance (GE) or Allegro (Philips)	5-10 MBq/kg or 5.18 MBq/kg	60 min
This study	Stage 3/4<br>Stage 1/2	34<br>7	5.48 (1.41–12.94)<br>2.61 (1.44–4.05)	Discovery ST-16 (GE)	5 MBq/kg	45 min
Lee *et al*. [29]	Stage 4<br>Stage 1/2/3/4S	31<br>19	5.2 ± 2.8<br>2.5 ± 1.3	GE Advance (GE) or Allegro (Philips)	5 MBq/kg or 5.18 MBq/kg	60 min
This study	Stage 4<br>Stage 1/2/3/4S	25<br>17	5.7 ± 2.8<br>3.8 ± 2.7	Discovery ST-16 (GE)	5 MBq/kg	45 min

*Mean (range) or mean ± standard deviation.
†Injection-to-acquisition time.

REFERENCES

15. Choi YJ, Hwang HS, Kim HJ, Jeong YH, Cho A, Lee JH, Yun M, Lee JD, Kang WJ. (18)F-FDG PET as a single imaging modality in pediatric neuroblastoma: comparison with abdomen CT and bone scintigraphy. Ann Nucl Med. 2014; 28:304–313.

29. Lee JW, Cho A, Yun M, Lee JD, Lyu CJ, Kang WJ. Prognostic value of pretreatment FDG PET in pediatric neuroblastoma. Eur J Radiol. 2015; 84:2633–2639.
